# The relationship between accessibility of retail seeds and diet diversity: a multi-level structural equation model applied to ethnic minority farmers in northern Vietnam

**DOI:** 10.1007/s12571-025-01613-w

**Published:** 2025-10-21

**Authors:** Lan Thuy T. Nguyen, Marrit van den Berg, Jacob Bob Douma, Tjeerd Jan Stomph

**Affiliations:** 1 Development Economics Group, Wageningen University and Research, Hollandseweg 1, Wageningen, KN 6706 Netherlands; 2 Bioversity International, Penang, Malaysia; 3 Centre for Crop Systems Analysis, Wageningen University and Research, Wageningen, Netherlands

**Keywords:** Seed accessibility, Seed systems, Crop diversity, Seed procurement methods, Impact pathways

## Abstract

Undernutrition remains a critical issue among ethnic minorities in northern Vietnam, likely due to limited diet diversity. Our study examined the accessibility of retail seeds, its link to diet diversity, and the relative contribution of food and seed market access and income on diet diversity, specifically targeting diversity in vegetables and legumes. We utilized a comprehensive dataset, comprising a household survey conducted with nearly 500 respondents, a community survey in 38 villages, and a market survey of 14 marketplaces. Using the multi-level structural equation modelling (MSEM) method, we empirically validated a conceptual model linking diet diversity with food and seed market access and satisfaction with off-farm seed sources, where the latter two are aspects of accessibility of retail seeds. The linkages are mediated by crop diversity at both village and household levels, and household wealth. Results showed that retail seeds played a key role in enhancing crop diversity, while self-saved seeds remained the dominant seed source. Crop diversity influenced diets directly through the subsistence pathway and indirectly through the income pathway, though the latter effect was smaller. Household wealth also improved diet diversity directly and indirectly by promoting crop diversity, suggesting that income sources outside of vegetable and legume production are crucial for supporting diverse diets. Market access enhances diet diversity through food purchases and indirectly through crop diversity, yet this indirect effect is much smaller. Its overall impact on crop diversity was more nuanced due to multiple influences. Furthermore, farmers’ satisfaction with off-farm seed sources indirectly improved diet diversity by increasing crop diversity. Our findings suggest that promoting crop diversity and supporting food purchases are both essential for improving diets. Encouraging integrated seed systems that recognize the role of both farmer and formal seed systems could further enhance crop diversity and diet diversity.

**Supplementary Information:**

The online version contains supplementary material available at 10.1007/s12571-025-01613-w.

## Introduction

Undernutrition is a serious global health problem, especially in low and middle-income countries (LMICs) (FAO et al., 2023), including Vietnam. In recent decades, Vietnam has made remarkable progress in reducing undernutrition, and over-consumption has become the key challenge, especially in urban areas (Chaparro et al., 2014; Cuong et al., 2007). However, ethnic minority groups living in remote mountainous areas are still struggling with chronic undernutrition (Mbuya et al., 2019). A study on pre-school children in Northern Vietnam found that ethnic minorities had a malnutrition rate twice as high as the majority group (Le et al., 2019). Undernutrition is influenced by multiple factors, including water sanitation, care practices, and health access. One of the major contributing factors to undernutrition in these communities is their starch-based diet, with a limited number of sources of micronutrients (Eckhardt, 2006; Nguyen et al., 2013).

Out of the various elements contributing to malnutrition, this paper focuses on the potential to enhance dietary diversity as a contribution to improving nutrition. Promoting the consumption of diverse vegetables and legumes is one of the promising strategies for improving micronutrient intake, and hence addressing micronutrient deficiency (Duthie et al., 2018). These foods are foundational to a nutritious diet, rich in vitamins, minerals, proteins, and dietary fiber, which, in addition to energy, are essential for overall health and growth. The nutrients found in vegetables and legumes play a vital role in supporting the immune system, preventing stunted growth, and fostering cognitive development (Polak et al., 2015; Slavin & Lloyd, 2012). The World Health Organization (WHO) recommends that adults eat at least 5 portions, or 400 g, of fruits and vegetables daily for protective nutritional effects (WHO, 2003). National food-based dietary guidelines further interpret this advice, suggesting the intake of multiple portions of a variety of fruits and vegetables each day (Herforth et al., 2019).

Crop diversification is a promising avenue to achieve diet diversity, especially in the context of ethnic minority communities in Vietnam, who often depend on agriculture for both their food and their livelihood. The rationale behind this is based on the intuition that increasing the variety of crops cultivated could have a positive impact on diet diversity via direct consumption of the increased diversity at the farm level (subsistence pathway), or improved purchasing power thanks to greater income from production sales (income pathway). Besides, diversification efforts can be constrained by limited seed accessibility, as seeds form the foundation of any agricultural system. Nevertheless, little is known about the role of seed accessibility in the linkage between agriculture and nutrition, especially the role of nutrient-dense crops such as vegetables and legumes.

Our study focuses on the accessibility of retail seeds for farmers in the local markets. Self-saved seeds play a vital role in supporting smallholder farming systems, particularly by ensuring timely access to planting material and maintaining genetic diversity within crop types that are often well-adapted to their farms’ conditions (McGuire & Sperling, 2016). Nevertheless, relying on self-saved seeds alone could limit farmers’ access to new or underutilized crop types^1^. On the other hand, the local seed market provides an effective avenue for farmers to exchange seeds both via formal and informal systems (Lipper et al., 2010). It complements on-farm seed systems by providing access to a broader range of species and varieties (Lipper et al., 2010; Nguyen et al., 2026). Therefore, we consider the accessibility of retail seeds as essential to support diversification strategies.

In a rural context, the retail seed market cannot be studied in isolation from broader market access. This is because various types of markets, whether for seeds, food, or agricultural inputs and outputs, are typically integrated into a single local marketplace. This integrated nature means that farmers’ ability to obtain retail seeds is often intertwined with their access to food, input, and output markets. Thus, understanding the accessibility of retail seeds inevitably leads to questions about overall market access.

Against this backdrop, we seek to answer the following three research questions. *Question 1*: How accessible are vegetable and legume retail seeds to ethnic minorities in northern Vietnam? *Question 2*: Via which pathways does accessibility of retail seeds affect diet diversity? *Question 3*: What are the relative contributions of market access and crop diversity to diet diversity? These questions were answered by analyzing a comprehensive dataset, which included a household survey of nearly 500 ethnic minority respondents from 38 villages in northern Vietnam, a village-level survey, and a market survey conducted in July 2022.

To answer the second and third questions, we applied multilevel structural equation modeling (MSEM) (Heck & Thomas, 2015). This method not only helps correct for dependencies among observations through the hierarchical sampling design but also explicitly investigates the contributions of variables that change at higher aggregation levels (e.g., villages) to outcomes at lower aggregation levels (individual households). It does so by testing whether the hypothesized cause-and-effect structure among the variables is consistent with the patterns of covariation in the data.

We contribute to the literature in several ways. First, to the best of our knowledge, our study is among the first attempts to build empirical linkages between accessibility of retail seeds and nutrition, focusing on vegetable and legume food groups. Existing research has largely centered on the cultivar diversity of starchy staple crops, or indigenous, nutrient-dense crops (Bicksler et al., 2012; Croft et al., 2018; McGuire & Sperling, 2016). However, enhancing diet diversity requires attention to a broader range of food groups and the nutritional value of both indigenous and introduced non-staple crops.

Second, we bring the context of ethnic minorities into the ongoing debate on whether crop diversity or market access and income play a greater role in improving diets. While many studies show positive links between farm diversification and diet diversity, others argue for the primacy of market access and income (Sibhatu et al., 2015; Sibhatu & Qaim, 2018). Markets can expand diet options by connecting households to foods that they do not produce themselves. However, market access can complicate food choices and have uneven effects on different food groups. For instance, vegetables and legumes, the food groups traditionally sourced on-farm, may be traded off when households rely more on purchased food as observed in Indonesia (Mehraban & Ickowitz, 2021). The dynamics between crop, diet, and market could be different in the specific context of ethnic minorities, who frequently face barriers to market participation due to language and cultural differences (Fujii, 2018; van de Walle & Gunewardena, 2001). These structural constraints could shape how market access interacts with on-farm diversity to affect diet outcomes, underscoring the need for more context-specific evidence.

Third, our study contributes to the growing body of research examining the relationship between crop diversity and diet diversity across multiple scales. Mixed findings in the literature may be partly due to the emphasis on the household level (Fanzo, 2017; Tobin et al., 2019). For example, one study found no significant link between crop diversity and diet diversity at the household level, but a positive association at the landscape level (Remans et al., 2011). This suggests that households may benefit from broader agrobiodiversity shaping broader food environment, without necessarily cultivating a wide range of crops themselves. By facilitating exchange, local food markets can mediate access to a wider variety of crops, highlighting the importance of considering both household and landscape-level dynamics when assessing the relationship between crop diversity and diet diversity.

## Materials and methods

### Conceptual causal model

**Fig. 1:**
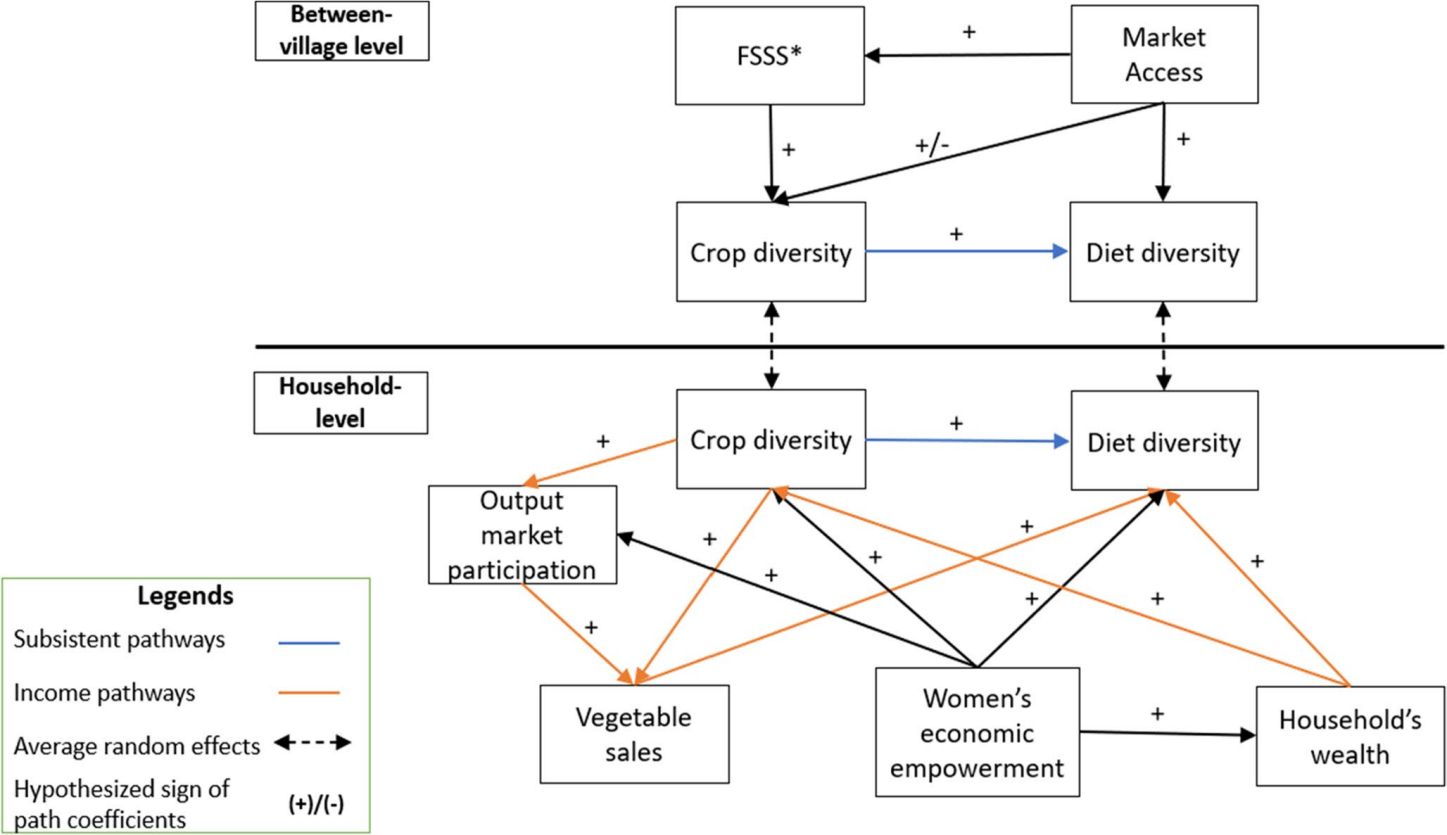
Hypothesized causal pathways from accessibility of retails seeds to diet diversity at the between-villages and household levels. Note: * FSSS: Farmers’ satisfaction with off-farm seed sources

Our conceptual model is represented by a directed acyclic graph (DAG) (Fig. 1). This graph consists of variables connected by directed arrows, which depict the suspected causal relationship between variables, and does not contain feedback loops between any two variables. A DAG facilitates clear communication of the causal structure, and allows for testing the direct and indirect pathways affecting the outcomes of interest (Digitale et al., 2022).

Our model comprises two components: the upper panel captures `between-villages’ level effects on diet diversity, and the lower panel, refers to `household’ level effects on diet diversity (Fig. 1). The upper panel depicts the connections of factors that vary at the `between-villages’ level and that affect crop and diet diversity at the `household’ level. Indeed, village-level patterns often influence household crop and diet diversity. Diet diversity is molded by culinary traditions and available resources, which can affect the variety of foods accessible to individual households (Ogunba, 2010). Similarly, the crop choice of households is likely to be influenced by their neighbors as they are exposed to similar cultivation culture, local climatic and physical conditions (Galeana-Pizaña et al., 2021).

This panel also outlines our core hypothesis linking the accessibility of retail seeds to diet diversity. Accessibility of retail seeds is defined as the hypothetical ability of farmers to obtain retail seeds. In the crop diversity context, this construct comprises three key elements: physical accessibility of retail seeds, availability of a diverse range of seeds at the market, and farmers’ satisfaction with the off-farm seed sources. In rural settings, input and food markets are often integrated into one physical space. This integration makes it difficult to isolate access to retail seeds from broader market access. In our study context, the availability of both diverse seeds and diverse foods tends to correlate with the size of the local market, reinforcing the idea that access to retail seeds is tightly linked to general market dynamics. Therefore, we define *market access* as the distance to local marketplaces, adjusted to the market size. This measurement captures the multiple roles of the market in facilitating access to both food and agricultural inputs (including seeds), thereby influencing *crop diversity* and *diet diversity.* Nevertheless, *market access* could entail the output market, which triggers the commercialization process, possibly leading to the retraction of *crop diversity*. Therefore, the total effect of *market access* on *crop diversity* can be either positive or negative, depending on the balance between input access and commercialization pressure (Fig. 1).

However, physical proximity alone does not guarantee meaningful access, especially if farmers find the seeds available at the marketplaces unsatisfactory. This perception is captured in the construct *farmers’ satisfaction with off-farm seed sources (FSSS)*, which reflects farmers’ subjective evaluation of how well a seed source aligns with their preferences. *FSSS* encompasses various dimensions, including trust in the vendor, availability of preferred varieties, affordability, and perceived quality of the seeds and the vegetables they produce. For instance, if farmers suspect that counterfeit or adulterated seeds dominate the marketplace, or if the varieties and crop types offered do not match their agroecological requirements or consumption preferences, they may search for another source (Kangile et al., 2018; Sarkar et al., 2024). In this case, farmers face access constraints when the lack of satisfaction overrides the advantage of proximity, highlighting the importance of understanding not just where seeds are sold, but how farmers perceive and experience these sources. *FSSS i*s measured as a collective rating to make this exogenous from household-level unobserved characteristics, as it reflects shared perceptions at the community level rather than individual household biases.

*Market access* also plays a role in shaping *FSSS*. Farmers living closer to a market often have greater exposure to seed purchasing options and information, which is crucial for building social trust (Zeleke et al., 2023). This factor is essential in informal seed systems, which primarily deal with uncertified seeds and involve transactions between farmers or between farmers and unregistered traders. These systems are often the main seed sources of smallholder farmers in developing contexts (McGuire & Sperling, 2016). As a result, farmers with better market access also have better chances of obtaining seeds that are well-suited to their needs, which may lead to higher *FSSS* (Subedi et al., 2008).

The lower panel was developed inspired by Schultz’s theory of traditional agriculture, which identifies the dual purposes of small-scale farming: providing food and generating income (Schultz, 1964). Consequently, *crop diversity* is posited to enhance *diet diversity* through two main pathways: subsistence and income. Firstly, subsistence farming enables households to directly consume a variety of crops grown at the farm level (subsistence pathways). Secondly, *crop diversity* enhances *vegetable sales* through *output market participation*, (income pathway). Crop diversity can allow farmers to take advantage of various market opportunities and mitigate the risk from market shocks, thus encouraging them to participate in the output market (Tugault-lafleur & Turner, 2011). One could also argue that crop diversification may increase *vegetable sales* because the synergistic effects of cultivating complementary crops can lead to a higher overall return per unit of land. This efficiency gain occurs when the addition of one crop does not reduce the yield of others, but enhances the overall resource efficiency (Pleasant & Burt, 2010). Furthermore, farmers with higher crop diversity can offer consumers a variety of choices in the output market, which then boosts their sales. Both technical efficiency and diverse market offers are captured through the indirect effect of *crop diversity* on *vegetable sales* to *diet diversity*.

Additionally, the income pathway can be influenced by other income sources beyond vegetables and legumes, which are captured by *household wealth.* This variable can be positively associated with *diet diversity* and *crop diversity.* Richer households possess greater purchasing power, enabling them to acquire a more diverse selection of foods at the market (Onah et al., 2022b). Additionally, wealthier households have larger farm sizes and higher financial capacity to purchase seeds and other agricultural inputs, allowing them to cultivate a wider range of crops, thus enhancing their crop diversity (Maru et al., 2022). *Household wealth* can also indirectly affect *diet diversity* through *crop diversity* (Fig. 1).

Both *crop diversity* and *diet diversity* are potentially affected by the level of *women’s economic empowerment*. Economically empowered women, defined as those who can influence the decisions on use of income generated by all productive activities in the family, are more likely to acquire a more diverse range of foods, benefiting both their households and themselves (Galiè et al., 2019; Onah et al., 2022b). These women can have a significant influence on agricultural production, affecting households’ crop choices and farming approaches. These tend to favor high biodiversity cropping systems, and the cultivation of nutrient-dense crops and medicinal herbs (Connors et al., 2023; De Pinto et al., 2020). Furthermore, as they will engage actively in income-generating activities, their households are more likely to participate in the output market. Additionally, *women’s economic empowerment* can indirectly increase crop and diet diversity by positively influencing *household wealth.* Economically empowered women tend to be more active in income-generation activities, which leads to higher accumulation of durable assets (Voronca et al., 2017). This, in turn, enhances their ability to invest in diverse crops and improve diet quality.

### Study context

Vietnam is a multi-ethnic country with 54 different ethnic groups. The largest of these is the Kinh (or Viet) ethnicity, comprising 85% of the total population (GSO, 2019). The Vietnamese government uses the term ‘ethnic minority’ to refer to all groups other than the Kinh majority. Except for the Hoa (ethnic Chinese), who are well-assimilated into Kinh’s culture, ethnic communities distinguish themselves from the majority and each other through unique languages and various distinctive cultural practices.

Ethnic minority communities represent the most economically challenged groups in the country, with 45% living below the poverty line compared to only 3.1% among the Kinh (Pimhidzai, 2018). Compared to their counterparts, they also encounter difficulties in accessing markets, education, and healthcare services (Bonnin & Turner, 2014; Karlidag-Dennis et al., 2020; Målqvist et al., 2013). These struggles are partly due to their geographical location in remote and mountainous regions with poorer infrastructure, compared to coastal and delta regions where the majority of Kinh and Hoa reside (Imai et al., 2011). Additionally, systemic social exclusion and biased government policy explain a notable part of the hardships faced by minorities (Baulch et al., 2007; Turner, 2022).

**Fig. 2:**
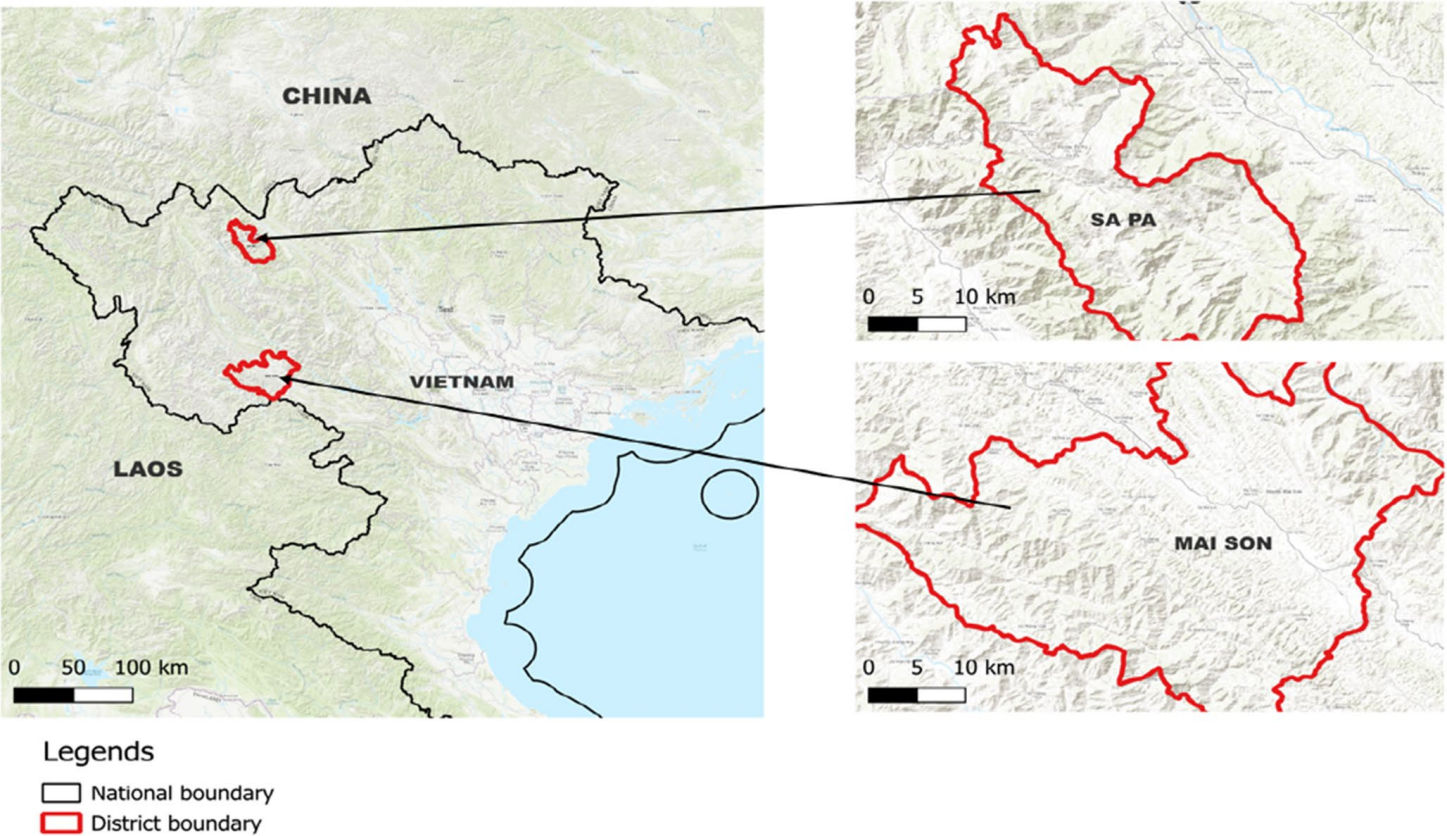
Study areas. In the right panel, the differences in shading indicates the difference in accentuation of the terrain in the studied districts

Our studied sites include 38 villages in five communes in two districts in mountainous northern Vietnam, the Mai Son and Sa Pa districts (Fig. 2). These communes are home to a substantial population of various ethnic minorities, including H’Mong, Thai, and Dao. The selected villages have climatic conditions highly favorable for horticultural activities throughout the year, with a wide range of diversity. Vegetables, along with rice, are considered a necessity in people’s diets in these regions. Most households maintain home gardens, and in addition often intercrop vegetables, legumes, and other annual crops like maize and rice. While production is primarily for home consumption, selling surplus produce, ranging from small-scale to primary livelihood, is common in our study sites.

Daily, traditional open-air markets are present in every commune and district center. These markets serve as the central hubs where farmers can purchase a wide range of goods, including agricultural inputs such as vegetable and legume seeds, food items, and household necessities. In addition, smallholder farmers can sell their produce at these markets, though typically in small quantities. Large-scale trading is usually handled by wholesalers, who often purchase directly at the farm gate. Some village clusters also host smaller informal markets, which offer a more limited selection. Besides fixed vendors, seasonal sellers, often local farmers, add to the diversity and accessibility of goods.

### Data collection

This study draws on two rounds of household survey data and a complementary seed accessibility survey conducted in 2021 and 2022 across 38 villages in the study regions. The household sample includes 494 women surveyed in July 2022, where 10 to 30 women were randomly selected per village from household lists provided by the local government. They were asked for detailed information on household demographics, the diversity of vegetables and legumes cultivated over the past three months, sources of the seeds for each crop, and the vegetable and legume consumption over the previous seven days. An earlier round of the same survey, conducted between December 2021 and March 2022 with a random sample of 636 women, is used to assess the robustness of the main results.

Although both rounds targeted the same respondents using a consistent questionnaire, the second round forms the basis of our main analysis for two reasons. First, the earlier round was interrupted by a COVID-19 outbreak, causing a two-month delay in data collection that led to the survey likely spanning two different agricultural and diet seasons, potentially affecting consistency in crop and diet diversity data. Second, the reduced number of completed cases makes longitudinal analysis not feasible. Third, we do not have longitudinal data on seed accessibility, as it was collected once, during the same time as the second survey.

In addition to the household survey, we conducted a community-based seed accessibility survey in July 2022. In each village, a separate group of 5 to 7 community members (excluding household respondents) who are knowledgeable about the seed procurement behavior of the village participated. A total of 194 respondents, of whom 65.5% were women, were recruited. The group questionnaire requested information on all venues/sources from which villagers typically purchased vegetable and legume seeds, and an assessment of farmers’ satisfaction with each of these sources. We then geo-referenced and assessed all 13 sources identified in the community survey, each of which also served as a local food and output market.

It should be noted that the household surveys were originally implemented as part of a broader randomized controlled trial that included training and seed support activities, but this study does not focus on evaluating treatment effects. The seed provision intervention was only a one-time event and is unlikely to influence current production, except potentially through self-saved seeds. This could potentially have changed interest in seed from the market, where the seeds were procured, or has generated interest in self-saving these seeds. However, we also found no significant treatment effects on crop or diet diversity in either survey round (Table 1, Appendix 2). A mixed-methods impact study found little evidence of this intervention on seed procuring behavior (Nguyen et al., 2025). The seed provision was excluded from farmers’ assessment of seed source, since the respondents were not seed recipients, making it difficult for them to evaluate the provided seeds reliably. Even if they had responded, their assessments could be biased due to the Hawthorne effect (McCarney et al., 2007).

### Measurements

#### Household level

Household-level indicators include *diet diversity*, *crop diversity*, *output market participation*, *vegetable sales*, *women’s economic empowerment*, and *household wealth*. To be specific, the *diet diversity* indicator is the total number of vegetable and legume types consumed by adult women in the household in 7 days preceding the interview. *Crop diversity* is the number of vegetable and legume crop types grown by households in the 3 months preceding the interview. Both indicators focus on crops that fall into one of four food groups according to FAO’s classification: 'dark green leafy vegetables’, ‘other vitamin A-rich vegetables’, `other vegetables’, and `legumes’ (or pulses) (FAO, 2021). The FAO includes fruits in 'other vitamin A-rich vegetables’. However, we excluded them from our analysis because the accessibility of fruit seeds (perennials) differs notably from that of vegetables and legumes (annuals). In the context of our conceptual model, these two indicators are treated as two parts: one part varies within villages, and another captures the remaining variation between villages.

*Output market participation* is a dummy variable indicating whether, in the past three months, the household grew at least one vegetable and legume crop with sale as a primary or secondary purpose, or if they had positive earnings from vegetable and legume sales in the month preceding the interview. *Vegetable sales* was determined by the farmer’s self-reported revenue from selling vegetables and/or legumes. To measure the *household wealth*, we extracted the first component produced by a principal component analysis (PCA) of a list of durable assets and big livestock, including cows, buffalo, and horses. In our study context, these animals are considered assets that show wealth status rather than merely farm animals.

*Women’s economic empowerment* was measured using the scale from a Pro-WEAI survey, which has been validated in 13 countries in Africa and Asia (Malapit et al., 2019). For each agricultural and non-agricultural activity in which the respondents participated, they were asked the extent of their contribution to the decisions relating to the output and income generated from this activity, using a 3-Likert scale, from (1) 'Not at all’ to (3) 'To a high extent’. The respondent is considered to have control over the use of income if they contribute at least to some extent on decisions on income from all activities they participate in (Seymour et al., 2023). A sample of the questionnaire can be found in Appendix 1.

#### Between-villages level

The *market access* of a village is calculated as the reciprocal of average distances to all marketplaces in which farmers can buy food and seed, where distance is corrected for the market size, and can be obtained by the following formula:

$$\:\mathrm{MA}=\mathrm N\ast\frac1{\sum\:_{\mathrm N}^{\mathrm i=1}{\mathrm{Constraint}\:\mathrm{factor}}_{\mathrm i}\ast{\mathrm{Distance}\:\mathrm{to}\:\mathrm{the}\:\mathrm{market}}_{\mathrm i}}$$

In which:

$\:MA$ Market access of a village.

$\:N$ The number of food and input marketplaces that villagers usually go to acquire food and/or vegetable and legume seeds within the study districts. A marketplace should have at least one vendor selling various types of fresh food and seed items.

$\:{Constraint\:factor}_{i}$ was determined based on the classification of markets into big, medium, and small across two study sites. The market has the discount factor 1 if it is a big market, 2 if it is a medium market, and 3 if it is a small market. A market is a `big market’ if it has more than 5 seed vendors and 5 food vendors, a `medium market’ if it has 3–4 seed vendors and 3–4 food vendors, and a ‘small market’ if it has 1–2 seed vendors and 1–2 food vendors.

$\:{Distance\:to\:the\:market}_{i}$ was measured by the Euclidean distance between the GPS coordinates of the market and the village center.

*FSSS* was measured as the average score assigned by villagers to each off-farm seed source they commonly used for procuring seeds. Community members were asked to rank their satisfaction with seeds from all off-farm sources on a scale from very low (1) to very high (5). The off-farm seed sources are marketplaces at district, commune, or village levels.

*Crop diversity* and *diet diversity* were measured as the random intercepts at the `between-villages’ level produced after fitting these variables at the `household’ level. it should be noted that since the two variables are at the village level, they can explain the mean crop or diet diversity `between-villages’ level, but not the variation in crop and diet diversity within a village (or `household’ level). Hence, there are no causal paths from the `between-villages’ to the `household’ level.

### Data analysis

#### Multilevel structural equation model (MSEM)

In this study, MSEM is particularly well-suited for analyzing the complex interactions within our data and aligns with our study’s objectives. Traditional analytical approaches, such as univariate and bivariate analyses, often limit the examination to one or a few linear relationships at a time. Structural equation model (SEM) provides a more robust framework by enabling the analysis of multiple relationships simultaneously and allowing us to disentangle the direct and indirect causal effects of variables on each other. Over the last two decades, SEM has gained widespread recognition and been substantively applied in various academic disciplines, including economics, sociology, ecology, and psychology.

The integration of multi-level modeling with SEM, known as MSEM, further enhances the SEM method by accommodating different hierarchical structures. This method was typically developed to address hierarchical data structures, for example, households nested in villages, as in our study sample. MSEM also allows us to explicitly disentangle and analyze effects at different levels of the data structure and to avoid the Simpson’s paradox, providing a more detailed understanding of the interactions and dependencies of each factor at each level (Sprenger & Weinberger, 2021).

#### Model testing and hypothesis testing strategy

We began our analysis by examining the intra-cluster correlation (ICC) of diet and crop diversity to verify the necessity of the multi-level structure. The ICCs of the two indicators were relatively high (> 0.25), which confirms our choice of model structure (Appendix 2- Table 2). We then fitted the conceptual model (Fig. 1) using Mplus’ MLR estimator (Muthén & Muthén, 1998–2017) on the data of the survey round in July 2022 (survey round 2). This approach helps to address potential non-normality issues in our data, which provides robust point estimates and robust goodness of fit indices and adjusts for the skewness and kurtosis of data distributions. Furthermore, our model estimation is conducted using all available information, a full information maximum likelihood approach (FIML). This method is generally more robust than methods relying solely on completed cases (Heck & Thomas, 2015). Next, we evaluated the goodness of fit of the model. Details on the evaluation of the model fit are provided in the following sections. As the model fit was satisfactory, we proceeded with a series of analyses to address our research questions. After fitting the model using the second round, we validated this model using the first round.

Apart from assessing the causal model, we assessed the effect of *FSSS*, a key aspect of accessibility of retail seeds, on *diet diversity* to answer the second research question. The third research question is answered by the direct and indirect effects of *crop diversity* and *market access* on diet diversity at the ‘household’ and ‘between villages’ levels. These effects were computed from the two fitted models using the second round and reevaluated with the first round.

Additionally, we tested the robustness of all analyses above by replacing the indicated *distance to market* with a weighted distance by village. This decision was motivated by the fact that our measurement of *distance to market* could incorrectly reflect respondents’ true travel costs since it simply measured the shortest Euclidean distance by GPS coordinates without correction for terrain or road conditions. Meanwhile, compared to Mai Son, Sa Pa has more rugged terrain (cf. Figure 2) characterized by steep and high mountains, and poorer road conditions. Thus, with the same distance measured by GPS coordinates, the travel time in Sa Pa could be higher than in Mai Son. Therefore, in these robustness checks, each village’s *distance to market* was weighted based on the reported travel time by foot to the nearest district market per village. This assumed that the calculated weight was equally valid for all markets, not only the district market. The details of how the weights were constructed are presented in Appendix 4.

#### Evaluation of fit

We employed the chi-square test statistic to assess the overall model fit. However, some scholars express concern that the Chi-square is sensitive to sample sizes, in which small sample sizes may lead to insufficient power to reject the hypothesized causal structure (Wang & Wang, 2012). Therefore, we crosschecked the results with four other popular statistics to evaluate the goodness of fit, which are root mean square error of approximation (RMSEA), standardized root mean square residuals (SRMR) with values less than 0.08 (acceptable) or 0.05 (good), and the comparative fit index (CFI) and Tucker-Lewis index (TLI) with a value larger than 0.90 (acceptable) or 0.95 (good) (Hu & Bentler, 1999).

### Ethics

The randomized control trial study, which this research was part of, received ethical approval from Hanoi University of Public Health, IRB No 482,428/2020/YTCC-HD3, issued on 4th December 2020, and from Wageningen University and Research, issued on February 1, 2021. Before data collection, we obtained oral informed consent from each respondent. Respondents were informed about the study’s purpose, confidentiality, rights of respondents, reimbursement, and contact numbers.

## Results

### Characteristics of the respondents

On average, our sample includes women who were around 40 years old and typically had not pursued education beyond the secondary level. Households generally consisted of about 5.5 members. Women’s economic empowerment is less common in the study regions, as the proportion of households with a female having some control over income was 26% (Table 1). The wealth index has a mean of −0.13 (SD = 1.85), suggesting that, on average, households in our sample own few durable assets. This indicates that the sample is relatively poor. On average, each household earned $US75 in the month preceding the interview from vegetable and legume sales with a large standard deviation compared to the mean (SD=$1,087). This issue reinforced our choice of Mplus’ MLR estimator to fit the conceptual causal model. To further address the skewness, we log-transformed the income variables in our analysis.

**Table 1:** Characteristics of respondents

Indicators	Mean (SD)
Respondents finished secondary school	0.21 (0.41)
Age of respondents	39.4 (11.4)
Household size	5.50 (1.78)
Wealth index	−0.13 (1.85)
Women’s economic empowerment	0.26 (0.44)
Participating in the vegetable and legume market	0.27 (0.44)
Household income from vegetables and legumes in the month preceding the interview ($US)	75 (1,087)
Number of vegetable and legume species grown in the 3 months preceding the interview	7.23 (3.18)
Number of vegetable and legume species consumed in the 7 days preceding the interview	4.99 (2.44)
N	**494**

^a^*p*<0.1; *p < 0.05; **p < 0.01; *** *p* < 0.001

The skewness of income from vegetable and legume sales suggests that they were not the primary source of livelihood in our study regions. This is reinforced by our field observation that the main cash crop in Sapa was cardamom, while in Mai Son, it was sugarcane. Consequently, this supported our decision to exclude the link between *household wealth* and *income from vegetable sales* in our conceptual model (Fig. 1).

### Seed procurement and accessibility of retail seeds

Our results showed that farmers maintain a high level of vegetable and legume diversity, cultivating an average of 7.23 (SD = 3.18) crop types over the three months preceding the interview. While self-saved seeds remain the primary source, the markets play a crucial role as additional seed sources in supporting crop diversity for our study group (Table 2). 17% of the total number of vegetable and legume types grown were from seeds purchased from the market. The influence of the market remained substantial across different food groups, with the most important role observed in the *legumes* food group, with 20% of the crops grown with purchased seeds, and the least important role captured in *other vitamin A-rich vegetables* at 13%.

**Table 2:** Number of vegetable types and legume species grown in the past three months at the household level and proportion of crops from various procurement methods

Indicators	Total (Mean (SD)	Self-saved seeds	Purchased seeds	Other sources/Don’t know
Total	7.23 (3.18)	80%	17%	3%
Dark green leafy vegetables	2.99 (1.93)	81%	16%	2%
Other vitamin A-rich vegetables	0.46 (0.57)	87%	13%	1%
Other vegetables	3.00 (2.08)	83%	17%	0%
Legumes	0.62 (0.68)	77%	20%	3%

Table 3 presents farmers’ accessibility of retail seeds. In general, farmers had good physical access to seed sources. On average, they could purchase seeds from 2.0 (SD = 0.9) different marketplaces. Nearly all villages had access to two marketplaces that sell seeds, with an average distance of 6.3 km (SD = 4.5) to these marketplaces, and 3.7 km (SD = 4.4) to the closest marketplace. Farmers could travel as far as 51 km to buy seeds, although such trips are likely infrequent and combined with other market-related activities.

**Table 3:** Accessibility of retail seeds in terms of distance to nearest markets of different sizes, the number of legume and vegetable types available, and farmer satisfaction with seed from different markets

Variables	Mean (SD)	Min	Max
Number of off-farm seed sources currently used in each village	2.0 (0.9)	1	5
Distance from village center to all types of marketplaces (km)	6.3 (4.5)	0.7	18.5
To the closest marketplace	3.7 (4.4)	0.0	17.3
To the district market*	8.5 (5.7)	0.7	27.3
To the commune market	2.7 (2.1)	0.2	7.7
To the village market	1.7 (1.3)	0.0	3.5
Number of legume and vegetable types for which seed is available at the markets	53.7 (6.7)	25	63
District market (N = 4)*	51.5 (4.7)	45	55
Commune market (N = 5)	31.0 (11.5)	17	42
Village market (N = 4)	19.0 (15.7)	7	37
Farmers’ satisfaction levels with seed from all types of markets	3.8 (0.5)	3	5
District market*	3.8 (0.5)	3	5
Commune market	3.6 (0.8)	2	5
Village market	4.2 (0.4)	4	5

* One commune market in Mai Son province is classified as a district market because its size is notably bigger than other commune markets in our study regions

Marketplaces also offered a broad range of seeds. On average, 58.2 (SD = 4.9) different legume species and vegetable types were available in markets across villages. Furthermore, farmers found seeds from the market are of satisfactory quality, with an average score of 3.8 (SD = 0.5) out of 5. Among the different market types, village markets received the highest quality ratings (mean = 4.2, SD = 0.4), while commune markets received the lowest (mean = 3.6, SD = 0.8).

Besides, we observed significant variation in the accessibility of retail seeds across villages. Some farmers found the commune market to be an unsatisfactory seed source, others were completely satisfied with it as an off-farm seed purchasing option. In certain villages, farmers had to travel more than 17 km to reach the closest marketplace, over six times farther than the distance faced by villages with the shortest distance to the marketplace.

### MSEM parameter results

**Fig. 3:**
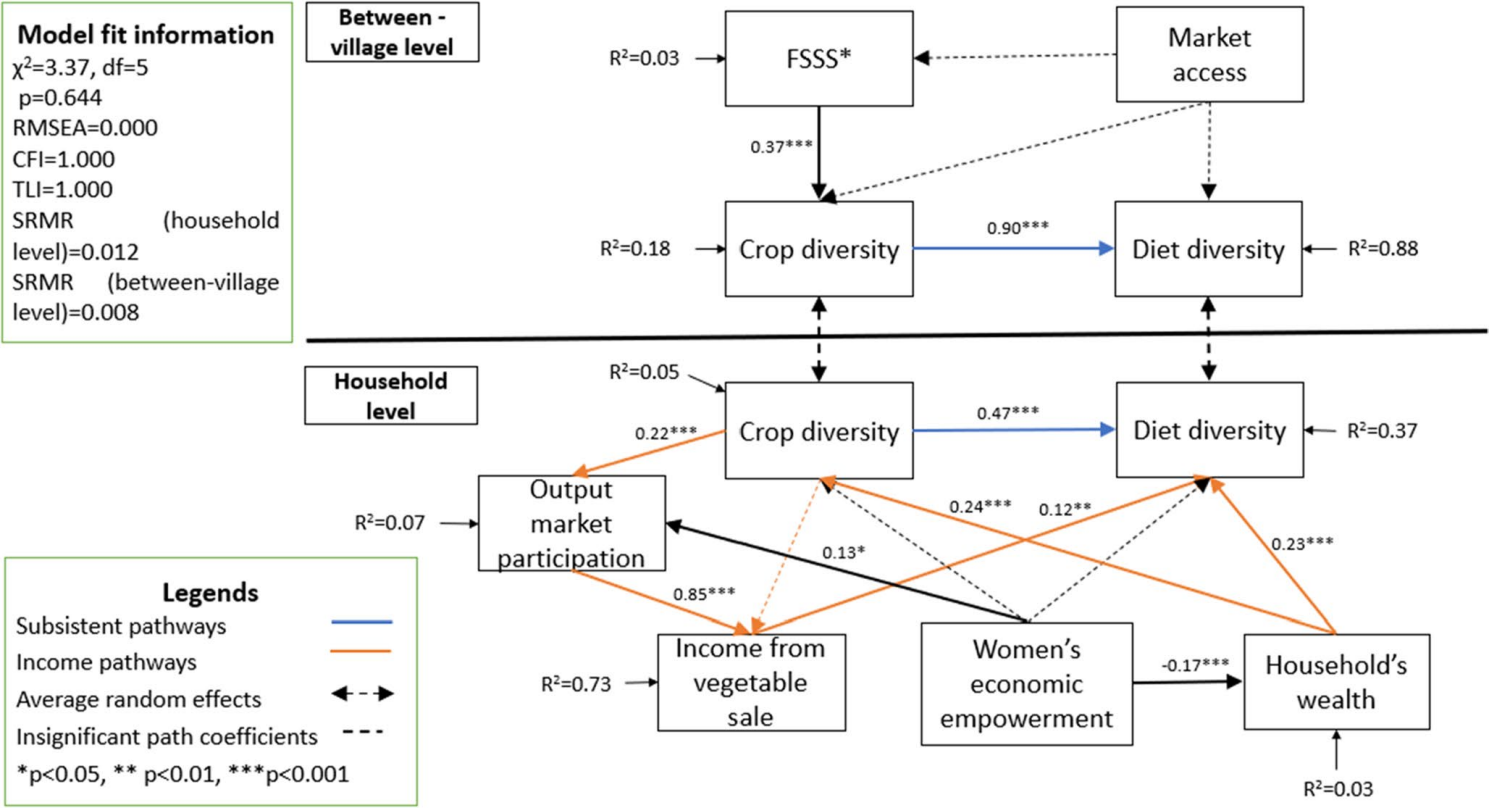
The structural equation model and standardized path coefficients. Note: * FSSS: Farmers’ satisfaction with off-farm seed sources

Figure 3 presents the parameters of our fitted model. The proposed causal model achieved a good fit as evidenced by the χ^2^ statistic (χ^2^ = 3.37, df = 5, p = 0.644). It means that our hypothesized DAG generates patterns of covariation that can reproduce those found in the data, although it does not exclude other possible DAGs that might be consistent with the data as well. The model’s adequacy is further supported by CFI and TFI as both are equal to 1.00, surpassing the threshold of 0.95, indicating an excellent fit between our conceptual model and the observed data. Additionally, the RMSEA is reported at 0.00, which is far below the recommended threshold of 0.05, further indicating a good fit (Hu & Bentler, 1999). SRMR provides insight into the model’s performance at different levels, showing a slightly better fit at the 'between-villages’ level (SRMR = 0.008) than at the 'household’ level (SRMR = 0.012). However, both SRMR values are well below the critical threshold of 0.05, suggesting a satisfactory reproduction of the variances and covariances among the variables at both levels. Our model effectively explains the variations in *diet diversity* at both 'household’ (R^2^ = 0.37) and ‘between villages’ levels (R^2^ = 0.88). The model also adequately accounts for the variations in *crop diversity*, especially at the ‘between-village’ level with R^2^ = 0.18 in the second round. Nevertheless, this variable was much less well explained at the household level (R^2^ = 0.06), which indicated that we may miss important factors.

Although the overall causal model adequately reproduced the observed covariance matrix, some path coefficients did not align with our hypotheses. Notably, we found a negative but non-significant direct effect of *women’s economic empowerment* on *diet diversity* (Fig. 3 & Appendix 4- Table 9), and its total effect was also insignificant (Appendix 4- Table 9). Similarly, no significant direct or total effect was observed between *women’s economic empowerment* and *crop diversity* (Fig. 3 & Appendix 4- Table 9). Moreover, *market access* showed no significant effect on *crop diversity*. This could be attributed to overall good access to diverse seed types in our sample, both from on-farm and the market. Besides, *market access* did not significantly influence *FSSS*.

### Direct and indirect effects of agriculture and market access on diet diversity

**Fig. 4:**
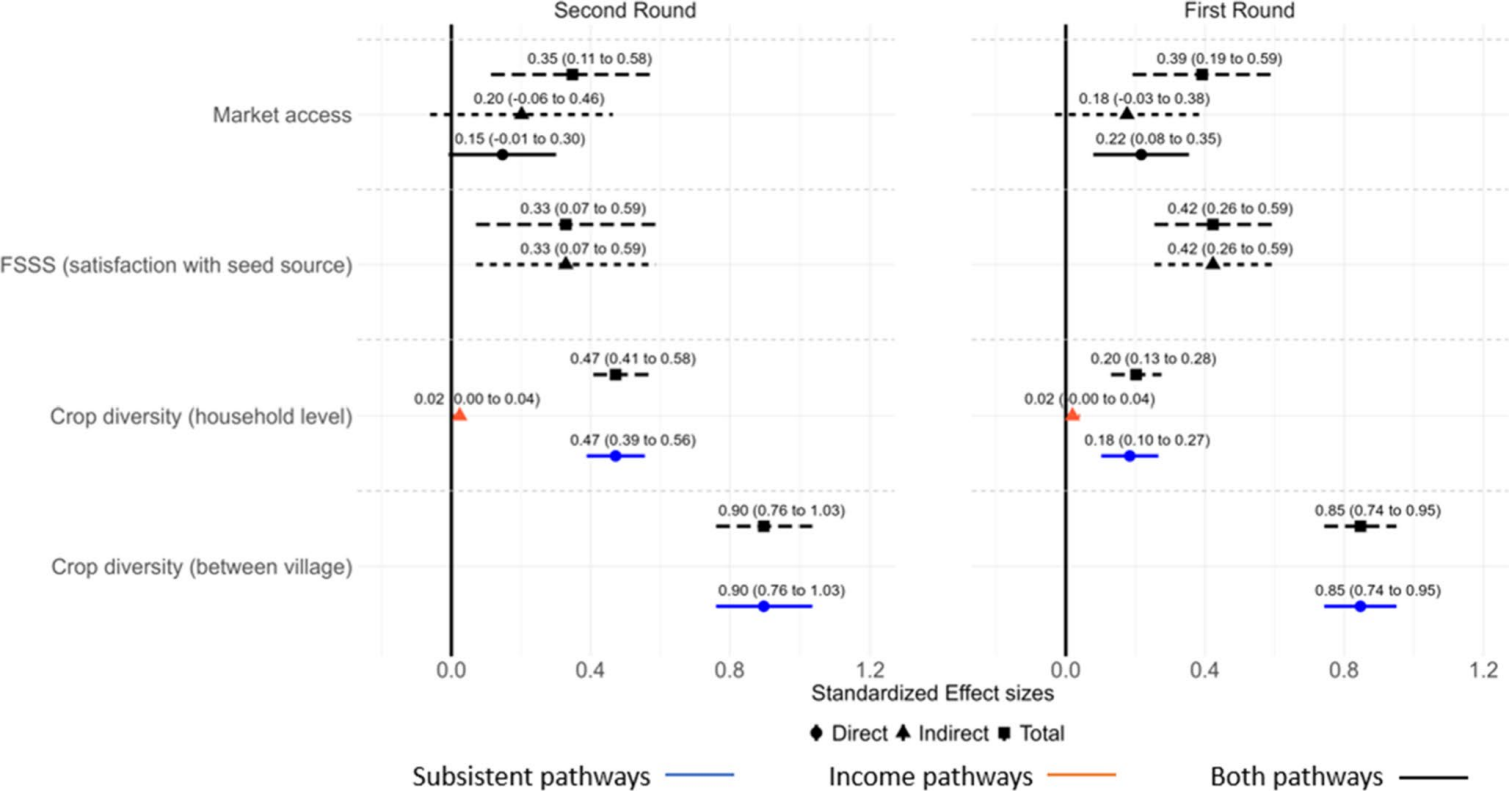
95% Confidence interval of standardized Total (squares and long-dashed lines), Direct (circles) and Indirect (triangles and short-dashed lines) effects of crop diversity, market constraints, and famers’ satisfaction with off-farm seed sources (FSSS) on diet diversity in two survey rounds. Note: Effect size estimated by using data from the market access indicator without correction for terrain (cf. Appendix 5- Fig. 4 for weighted market access)

Our analysis examined the standardized direct and indirect effects of *crop diversity*, *market access*, and *household wealth* on *diet diversity*. We found that *crop diversity* was positively associated with *diet diversity* primarily through the subsistence pathway. At the ‘between-village’ level, an increase of one standard deviation (SD) in crop diversity corresponded to a direct increase of 0.90 SD (95% CI = 0.76–1.03 SD) in diet diversity. At the ‘household’ level, the direct effect was 0.47 SD (95% CI = 0.39–0.56) (Fig. 4). This indicates that villages with greater crop diversity tend to have greater diet diversity, while this effect is less pronounced at the ‘household’ level.

We also found that the income pathway notably contributed to diet diversity, although this contribution was mostly from income sources other than vegetables and legumes. Specifically, one SD increase in crop diversity was associated with a 0.02 SD (95% CI = 0.00–0.04.00.04) increase in diet diversity (Fig. 4). Meanwhile, the total effect of *household* wealth on *diet diversity* was 0.27 SD (CI = 0.16–0.38 SD), with the direct effect being remarkably larger than the indirect effect via *crop diversity* (Fig. 4). This is in line with our previous result, implying that *income from vegetable sales* is not the key income source in our study context.

Additionally, increased *market access* can be positively associated with direct improvement of *diet diversity* but not the indirect effect. Specifically, one SD increase in *market access* was directly linked to a 0.15 SD (95% CI=−0.01-0.30 SD) increase in *diet diversity*. Besides, our findings suggest that most effects of *market access* were through the direct pathway (food purchase), while the indirect pathway through *crop diversity* (seed purchase and food market demand effect) was not significant.

Additionally, we found significant positive effects of *FSSS* indirectly via crop diversity (Fig. 4). Specifically, a one SD increase in *FSSS* is linked with 0.36 SD (95% CI = 0.13–0.59 SD) improvement in diet diversity. The significant effect of *FSSS* can be attributed to its dual influence through both subsistence and income pathways. Not only does *FSSS* affect diets through the subsistence pathway, but it also enhances income gained from increased *crop diversity*, which in turn contributes to *diet diversity* (Fig. 4).

### Robustness checks

We conducted robustness checks on the conceptual causal model using two complementary strategies. First, we fitted the model using data from the first survey round, conducted between December 2021 and March 2022. Second, we recalculated the *market access* variable using village-level weighted *distance to market* (Appendix 3). The model based on the first survey round met all model fit criteria (χ2 = 2.70, df = 5, *p* = 0.746, RMSEA = 0.000, CFI = 1.000, TLI = 1.000, SRMR (household level) = 0.013, SRMR (between villages level) = 0.020) (Appendix 4 -Fig. 3). Particularly, the path coefficient and total effect of *women’s economic empowerment* on *diet diversity* were significantly negative, confirming the inverse (negative) relationship between the two constructs (Appendix 4- Fig. 3; Table 9). When using weighted *market access*, the results remained consistent across both the first and the second round (Appendix 4 – Figs. 3 and 4). We also used data from the first round to examine direct and indirect effects of *market access* and *FSSS* on *crop diversity* and *diet diversity*. The direction of effects was consistent, although effect sizes varied (Fig. 4). These patterns held when using the weighted *market access* in both survey rounds (Appendix 5- Fig. 4).

## Discussion

This study investigated the role of accessibility of retail seeds on the linkages from crop diversity to diet diversity, and the relative contributions of different pathways from crop diversity to diet diversity, in the context of ethnic minorities in northern Vietnam. We used a comprehensive dataset from a household survey and an accessibility of retail seed survey to first understand the accessibility of retail seed in the study context, and test a conceptual causal model (Figs. 1 and 3) using the MSEM method.

### Accessibility of retail seeds

Although self-saving is the primary seed source, the local seed market plays a complementary role in shaping on-farm crop diversity. Similar to other developing countries, the seed market in our setting operates within a loosely regulated environment (de Boef et al., 2025; Hunga et al., 2023). Field observations and informal conversations between the researchers and enumerators with seed vendors revealed significant uncertainty about seed origins and biological quality. The national regulatory framework does not mandate standardized quality testing or transparency regarding seed provenance (Kuhlmann et al., 2023). A review of the seed industry in 2014 found that imported vegetable seeds constituted approximately 80% of the seeds traded in the country without clear oversight (Dung, 2014). Our field observations showed many seed packages were labeled in foreign languages, reflecting their imported nature. While no newer data exists, unregulated imported seeds likely remain dominant, reinforcing the 'grey zone’ between formal and informal seed systems as described in earlier studies (CGIAR, 2020; SPER Market Research, 2024).

Despite expert concerns over counterfeit or substandard seeds, farmers reported broad satisfaction with the availability and quality of retail seeds (Barriga & Fiala, 2020; Gebeyehu et al., 2019). One explanation is that vegetable growing of most farmers in our study area is subsistence-oriented. Their evaluation is therefore less focused on strict technical standards and more on whether seeds meet everyday household needs (Urrea-Hernandez et al., 2016). Moreover, because our survey covered a wide range of crop types, farmers may have based their assessments on favorite crops with desirable consumption qualities rather than on technical performance across all crops. Their satisfaction may also be reinforced by the relatively low prices of retail seeds, which is partly due to weak regulatory enforcement, making the seeds more affordable for poor households and thereby encouraging the cultivation of diverse crops.

Although imported seeds raise concerns about national seed sovereignty, they provide essential resources for ethnic minority farmers. By filling the gaps left by the public and private seed sectors within the country, which have little incentive to serve remote populations, imported seeds expand crop options and complement locally produced seeds (Turner, 2022; Turner & Bonnin, 2018). In this sense, they contribute to the diversity of seed sources available to farmers, supporting broader crop choices. Our finding supports previous studies showing that access to a variety of seed sources is crucial for maintaining agrobiodiversity (Almekinders et al., 2021; Delaquis et al., 2024; Nguyen et al., (Nguyen, et al., 2026)).

### The linkages between accessibility of retail seeds and diet diversity

We found positive, significant roles of accessibility of retail seeds on *diet diversity* via *crop diversity*, reflected in the positive association of *FSSS* and *crop diversity*. This implies that when communities collectively value the retail seed sources, households are more likely to grow a wider range of crops, including nutrient-dense ones. This supports prior evidence that farmers’ perceptions of seed suitability to their needs and conditions are critical drivers of varietal adoption decisions (Maredia et al., 2019; Wossen et al., 2022). Our finding also speaks to the literature linking social trust and social learning with crop diversity. Although our study did not explicitly measure social trust or social learning, the collective method measuring farmers’ satisfaction may reflect the localized reputation of seed sources or communal learning about seed performance, the elements that are often embedded in informal seed systems (McGuire & Sperling, 2016; Pautasso et al., 2013).

Unexpectedly, we did not find a significant direct relationship between *market access* and *crop diversity*. This does not necessarily indicate that physical seed access is irrelevant to diet outcomes, but could be specific to our study context, where farmers generally reported good access to diverse seed types from all types of markets. The absence of an effect may also reflect competing incentives: enhanced access to input markets provides farmers with a broader range of seeds, while output markets can encourage specialization and monocropping to maximize income ((Montúfar & Ayala, 2019; Pingali & Rosegrant, 1995). These opposing forces may offset one another, explaining the null relationship.

Moreover, we observed no significant relationship between *market access* and *FSSS*, suggesting satisfaction depends more on perceived quality than proximity. Earlier studies found market distance negatively influence satisfaction as closeness to a market makes it more convenient and easier for trust-building, yet some farmers might favor more distant markets that offer greater variety or perceived quality (Ncube et al., 2023; Zeleke et al., 2023). These opposing preferences could produce effects in different directions, ultimately offsetting each other and resulting in the null relationship observed in our study.

Besides, the negative effect of *women’s economic empowerment* on *household wealth* and *diet diversity* diverges from earlier studies in Africa (Adebowale et al., 2016; Voronca et al., 2017). This may reflect our specific study context, where households with either men’s or women’s economic empowerment often have fewer income streams than those where both are disempowered (Appendix 4, Table 7). One explanation is that financial empowerment here can mean managing all income responsibilities alone, which may overwhelm individuals and reduce a household’s overall capacity (World Bank, 2019). Therefore, men and women typically take full responsibility of different income streams, leading to low empowerment for both. These dynamics could explain the absent link between *women’s economic empowerment* and *crop diversity*, and the potential negative association with *diet diversity*. Furthermore, the lack of a significant direct or total effect of *women’s economic empowerment* on *crop diversity* aligns with a recent review of grey literature, which suggests that decisions on crop production diversification are generally gender neutral, and another study in the same context found joint decision-making over the production domain is common (Anderson et al., 2021; DeJaeghere et al., 2022).

We confirmed the positive links between *crop diversity* and *diet diversity* at both the landscape (between village) and ‘household level’, with stronger effects at the ‘between village’ level. This implies that diet quality depends not only on what households grow, but also on broader local food environments shaped by communal practices, exchanges and markets. This underscores the importance of maintaining agrobiodiversity beyond the household level and aligns with earlier research emphasizing the need to examine agrobiodiversity beyond the farm scale (Remans et al., 2011; Tobin et al., 2019). Therefore, supporting vibrant, diverse local food environments could be essential to ensuring that all households have access to a healthy and varied diet.

### The relative contributions of market access and crop diversity on diet diversity

Both market access and crop diversity have positive influences on diets, but their relative importance varied. For vegetables and legumes, on-farm crop diversity has a larger effect than market access. Evidence from several African and Asian contexts suggests that crop diversification can sometimes compete with other livelihood activities, as it may divert resources that could otherwise support income generation (Koppmair et al., 2017). However, this conflict appears less relevant for vegetables and legumes, which can often be cultivated alongside other activities. In our study area, many vegetables require relatively little effort to maintain, reducing potential conflicts with other livelihoods. At the same time, markets remain essential for foods that are difficult to produce, store or process, such as legumes, which are more likely to be purchased in our study context and in other contexts in Africa and Asia (Onah et al., 2022a; Sibhatu et al., 2015).

### Study limitations and future research

Our study is subject to several limitations. First, we could not include the accessibility of self-saved seed in our conceptual model due to data structure and methodological constraints. The self-saved seed variable is derived from the same dataset used to construct household crop diversity, creating high collinearity and endogeneity. Self-saving is also influenced by cultural norms and ethnicity, which shape cropping and diet outcomes. Future work could address this using a more complex structural model or alternative identification strategies, such as experimental or quasi-experimental designs, which go beyond the scope of our current dataset.

Second, our model explained little of the variation in household crop diversity (R^2^ = 0.06 in the second round and R^2^ = 0.04 in the first round). Auxiliary analyses showed that household and individual characteristics did not explain vegetable and legume diversity, suggesting unobserved factors like crop preferences or overall diversification strategies may be more influential. Importantly, no additional tested variables were correlated with both crop and diet diversity, strengthening the robustness of our estimated direct effects.

Third, the limited number of clusters in our study restricted our ability to examine heterogeneity across seasons, ethnicities, or climatic regions. While some path coefficients differed between survey rounds, no consistent seasonal patterns emerged. This may reflect different cropping calendars across the three ethnicities, which obscured seasonal effects at aggregate level (Fig. 1- Appendix 2). Future research should design data collection strategies that capture seasonality more effectively. Comparative studies across cultural and regional contexts could also clarify how seeds, crops, diets and markets interact using multilevel, multigroup analysis.

Fourth, we were unable to disentangle the effects of different market types on crop and diet diversity. Rural markets are often integrated, making it difficult to separate their distinct influences on crop and diet diversity. Addressing this complexity would require alternative methodological approaches which are beyond the scope of this study and should be considered in future studies.

## Conclusion

Our study highlighted the importance of accessibility of retail seeds in enhancing crop diversity among ethnic minorities in northern Vietnam, which in turn plays a crucial role in improving diet diversity within these communities. Crucially, the application of a MSEM approach allowed us to unpack the complex, multi-pathway relationships between accessibility of retail seeds, crop diversity, and diet diversity. The MSEM proved its value in clarifying the mechanism through which direct factors, such as *market access*, *crop diversity*, *women’s empowerment*, and *household wealth*, contribute to diet outcomes. Furthermore, a multi-level approach revealed that crop diversity at both ‘household’ and ‘between village’ levels plays a role in shaping diet outcomes.

Beyond affirming the links between accessibility of retail seeds and diet diversity, our study contributes to the ongoing debate about the relative contributions of market access and crop diversity to diet diversity. Our model revealed that both factors are relevant and can operate simultaneously. Market access complements crop diversity in enhancing diet diversity, mainly via the food purchase pathway rather than via leveraging crop diversification. While the role of market access on diets is more straightforward, our study suggests a complex role of this factor on crop diversity, where multiple forces are introduced by different kinds of markets.

Our findings entail clear policy implications. Interventions aiming to improve diet diversity should not treat market access and crop diversity as mutually exclusive strategies. Furthermore, they should promote an integrated seed sector development that strengthens both formal and informal channels, ensuring farmers’ access to a diverse portfolio of high-quality seeds. Such systems can simultaneously support crop diversity and food purchases, ultimately enhancing diet quality for ethnic minority populations in northern Vietnam.

## Supplementary Information

Below is the link to the electronic supplementary material.

Supplementary Material 1 (DOCX 703 KB)

## Footnotes

1 Crop type follows the classification of food items as used by consumers. Each crop type has a distinctive name at the food market and its taste is distinguishable from other types. In taxonomy, this is at sub-species level for some and species level for others. Cabbages for instance have different types with names like cauliflower, broccoli or white cabbage, but botanically these are a single species. For each crop type many genetically different cultivars exist, a level of detail not considered here.
