# Supplementary material for: The relationship between accessibility of retail seeds and diet diversity: a multi-level structural equation model applied to ethnic minority farmers in northern Vietnam
Source: Food Secur. 2025 Oct 21;17(6):1571–88. doi: 10.1007/s12571-025-01613-w (PMC12756305; doi:10.1007/s12571-025-01613-w)
Supplement: Supplementary file 1 — Supplementary Material 1 (DOCX 703 KB) [file 12571_2025_1613_MOESM1_ESM.docx]

## **Appendix 1: Sample questionnaire to measure women’s control over income indicator**

**Code L2.04 and L2.05**

Little to no input in decisions …….1

Input into some decisions ………….2

Input into most or all decisions ….3

Not applicable / no decision made …….98

| Now I’d like to ask you some questions about your participation in certain types of work activities and on making decisions on various aspects of household life. | | **L201**: Did you [NAME] participate in [ACTIVITY] in **the past 12 months** (that is, during the last [one/two] cropping seasons), from this year? | **L204**: Do you join the discussion and contribute ideas about how much of the outputs of [ACTIVITY] to keep for consumption at home rather than selling? If yes, how much contribution do you normally make? | **L205:** Do you join the discussion and contribute ideas about how to use income generated from [ACTIVITY]? If yes, how much contribution do you normally make? |
| --- | --- | --- | --- | --- |
| **A** | Staple grain farming and processing of the harvest: (rice, maize) | Yes…...1  No…….2 🡪 ***activity b*** |  |  |
| **B** | Vegetable farming and processing of the harvest | Yes…...1  No…….2 🡪 ***activity c*** |  |  |
| **C** | Other horticultural crops and high value crop farming and processing of the harvest | Yes…...1  No…….2 🡪 ***activity c*** |  |  |
| **D** | Large livestock raising (cattle) and processing of milk and/or meat | Yes…...1  No…….2 🡪 ***activity d*** |  |  |
| **E** | Small livestock raising (sheep, goats, pigs) and processing of milk /or meat | Yes…...1  No…….2 🡪 ***activity e*** |  |  |
| **F** | Poultry and other small animals raising (chickens, ducks, turkeys) and processing of eggs and/or meat | Yes…...1  No…….2 🡪 ***activity f*** |  |  |
| **G** | Fishpond culture | Yes…...1  No…….2 🡪 ***activity g*** |  |  |
| **H** | Non-farm economic activities (running a small business, self-employment, buy-and-sell) | Yes…...1  No…….2 🡪 ***activity h*** |  |  |
| **J** | Wage and salary employment (work that is paid for in cash or in-kind, including both agriculture and other wage work) | Yes…...1  No…….2 🡪 ***activity i*** |  |  |
| **K** | Large, occasional household purchases (bicycles, land, transport vehicles) |  |  |  |
| **L** | Routine household purchases (food for daily consumption or other household needs) |  |  |  |

## **Appendix 2 : Treatment effects on crop diversity and diet diversity, and Intra-cluster correlation**

**Table 1:** Coefficients (SE) and significance levels of regressions between crop diversity and diet diversity indicators and the treatments in household surveys collected in July 2022

| **Variables** | **Treatment 1: Nutrition and agriculture training** | | | | | **Treatment 2: Seed provision** | | | | | |
| --- | --- | --- | --- | --- | --- | --- | --- | --- | --- | --- | --- |
|  | **Survey round 1** | | **Survey round 2** | | **Survey round 1** | | | **Survey round 2** | | |  |
|  | **Coefficients** | **p-value** | **Coefficients** | **p-value** | | **Coefficients** | **p-value** | | **Coefficients** | **p-value** | |
| Crop diversity | -1.63 (1.47) | 0.27 | 0.29 (0.65) | 0.66 | | 0.82 (0.50) | 0.11 | | -0.19 (0.35) | 0.60 | |
| Diet diversity | -0.23 (0.56) | 0.68 | 0.04 (0.59) | 0.94 | | 0.14 (0.17) | 0.42 | | 0.06 (0.30) | 0.85 | |

**Table 2:** Intra-cluster correlation

| **Variables** | **Survey round 1** | **Survey round 2** |
| --- | --- | --- |
| Crop diversity | 0.47 | 0.25 |
| Diet diversity | 0.47 | 0.39 |

## **Appendix 3 : Characteristics and farmers perceptions over bought and self-saved seeds of household survey collected in December 2021 to March 2022 (First round)**

**Table 3:** Mean (SD) Characteristics of respondents

| **Indicators** | **Mean (SD)** |
| --- | --- |
| Ethnicity |  |
| *Thai* | 33.4% |
| *H'Mong* | 54.7% |
| *Dao* | 11.9% |
| Respondents finished secondary school | 0.21 (0.41) |
| Age of respondents | 39.3 (11.4) |
| Household size | 5.51 (1.79) |
| Wealth index | -0.13 (1.85) |
| Female household member having control over income | 0.27 (0.44) |
| Participating vegetable and legume market | 0.37 (0.48) |
| Income from vegetable and legume in the past month per household ($US) | 208 (3,315) |
| Number of vegetable and legume species grown in the past 3 months | 8.25 (6.17) |
| Number of vegetable and legume species consumed in the past 7 days | 4.74 (2.44) |
| **N** | **636** |

**Table 4:** Number of vegetable and legume species grown in the past three months at the household level and proportion of crops from various procurement methods

| **Indicators** | **Total (Mean (SD)** | **% Self-saved seeds** | **% Purchased seeds** | **% Other sources/ Don’t know** |
| --- | --- | --- | --- | --- |
| Total number of vegetable and legume species | 8.25 (6.17) | 69% | 28% | 3% |
| *Dark Green Leafy vegetables* | 3.97 (2.71) | 71% | 26% | 3% |
| *Orange Fleshed vegetables* | 0.55 (0.83) | 77% | 20% | 3% |
| *Other vegetables* | 3.09 (2.91) | 69% | 32% | 0% |
| *Legumes* | 0.63 (0.85) | 50% | 34% | 16% |
| **N** | **636** |  |  |  |

**Table 5:** Percentage of respondents find the seeds at various marketplaces trustworthy

| **Statements** | **Percentage (N=636)** |
| --- | --- |
| Commune market | 62% |
| District market | 74% |
| Professional breeders | 58% |
| Friends and relatives | 72% |
| Contracting cooperative/input dealers | 48% |
| Self-saved seeds | 97% |

**Figure 1:** Number of crop types grown in the past three months and number of crop types consumed in the past 7 days by households of total sample and disaggregated by ethnicities. (*) denotes coefficients’ significance level of the regression between survey round and each indicator with standard errors clustered at village level. *** p<0.05, ** p<0.01, *** p<0.001

**Table 6:** Percentage of respondents agree with various statements on self-saving and purchasing seeds

| **Statements** | **Percentage (N=636)** |
| --- | --- |
| I like to save the seeds myself | 93% |
| In general, saving seeds is easy | 92% |
| I like to buy seeds at the market than saving seeds myself | 9% |
| In general, bought seeds are better quality than self-saved seeds | 23% |

**Table 7:** Coefficients and significance levels of the regressions between number of income streams and economic empowerment. Standard errors are clustered at village level

| **Independent variables** | **Coefficients** | **Robust SE** | **p-value** |
| --- | --- | --- | --- |
| Households with women’s economic empowerment | -0.62 | 0.17 | 0.00 |
| Households with men’s economic empowerment | -0.51 | 0.19 | 0.10 |

## **Appendix 4: Construction of weights for market access**

Given the concern that *distance to market* measured by Euclidean distance by GPS coordinates may not represent actual travel costs, we adjusted this variable by incorporating real travel time to the district market for each village. This data was gathered during the First Round by asking each household about their walking travel time to the nearest district market. We calculated the average travel time for each village. Subsequently, we determined the average travel speed for each village using the travel time and Euclidean distance between the village and the district market. Finally, we calculated the weighted market access using this formula:

$$MA_{weighted}=N*\frac{Speed}{\sum_{N}^{i=1} {Constraint factor}_{i}*{Distance to market}_{i}}$$

## **Appendix 5: Robustness check**

**Figure 2**: The structural equation model and standardized path coefficients presenting two theoretical impact pathways from seed availability and quality to nutrition using data from the **Second Round with weighted *market access***.


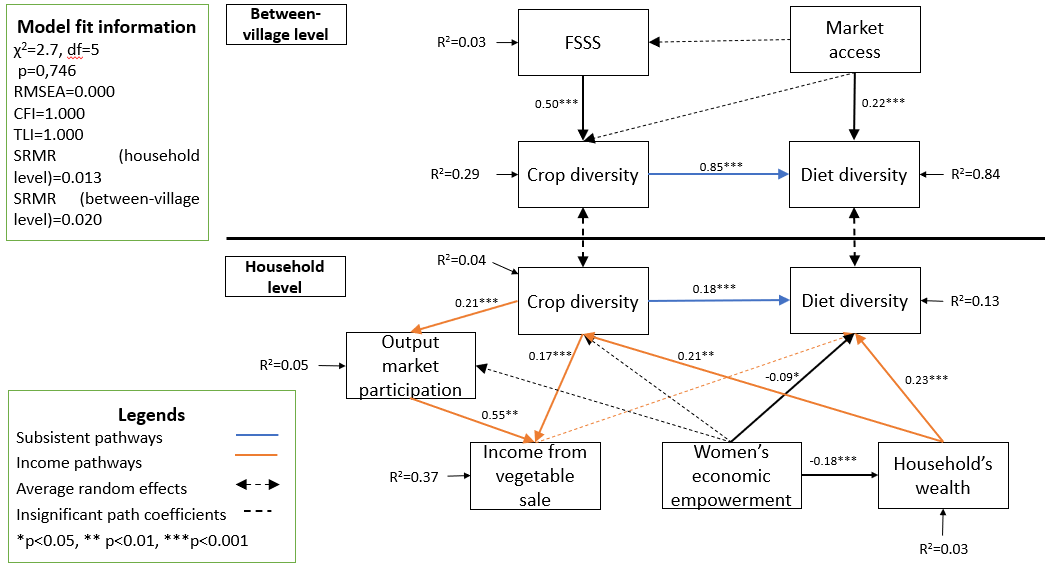


**Figure 1**: The structural equation model and standardized path coefficients presenting two theoretical impact pathways from seed availability and quality to nutrition using data from the **First round with unweighted *market access***.


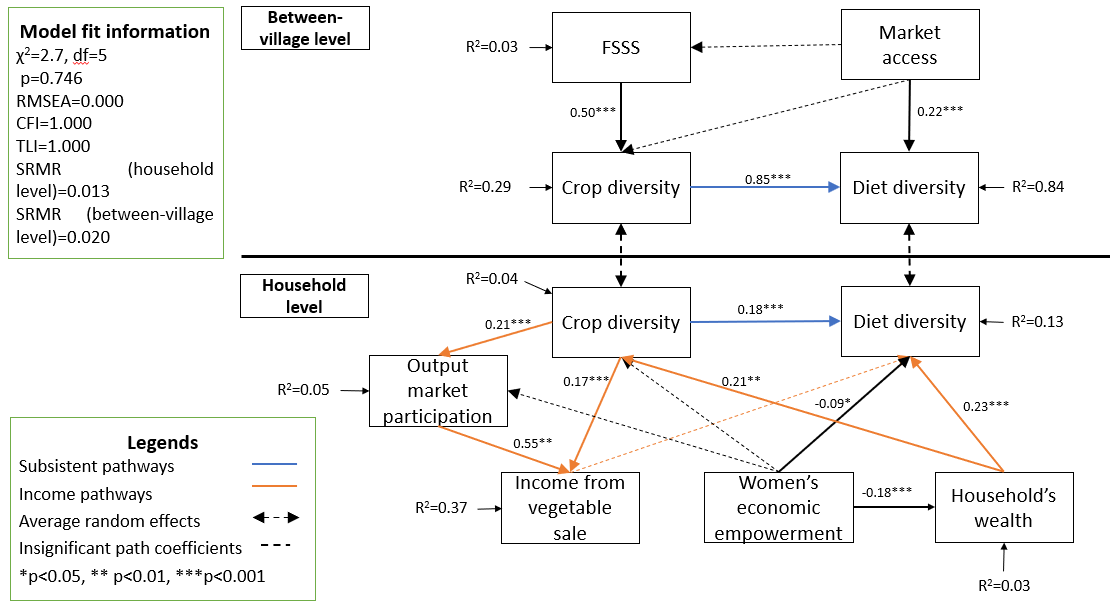


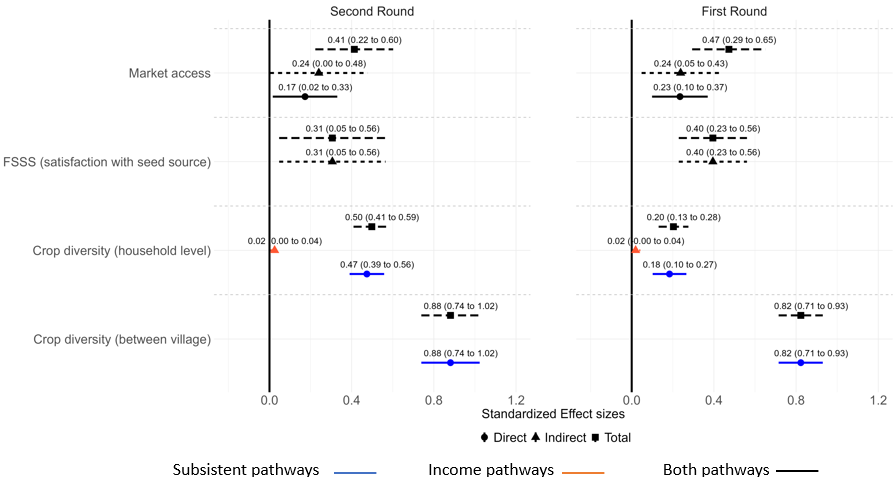


**Figure 4**: 95% Confidence interval of standardized Total (squares and long-dashed lines), Direct (circles) and Indirect (triangles and short-dashed lines) effects of crop diversity, market constraints and perceived seed quality on Diet diversity in two survey rounds – **Weighted market access**.

**Figure 3**: The structural equation model and standardized path coefficients presenting two theoretical impact pathways from seed availability and quality to nutrition using data from the **First Round with weighted *market access***.


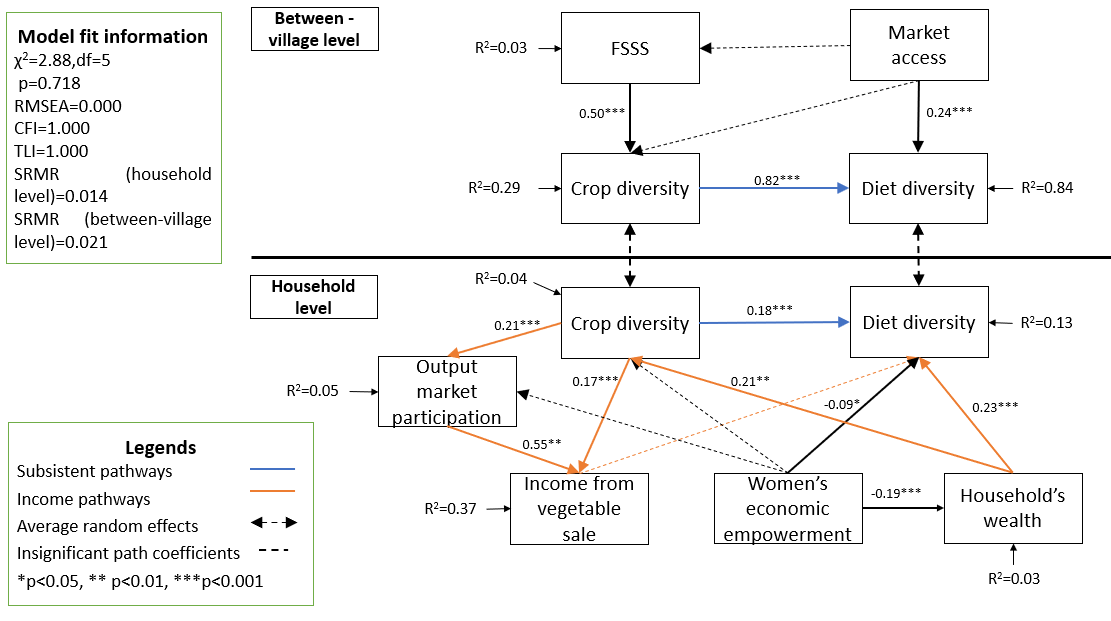


**Table 8:** Path coefficients (SE) of Crop diversity and Diet diversity, and their explanatory variables in a series of auxiliary structural equation models (*)

| **Explanatory variables** | **(1)** | | **(2)** | | **(3)** | | **(4)** | | **(5)** | | **(6)** | |
| --- | --- | --- | --- | --- | --- | --- | --- | --- | --- | --- | --- | --- |
|  | **Crop diversity** | **Diet diversity** | **Crop diversity** | **Diet diversity** | **Crop diversity** | **Diet diversity** | **Crop diversity** | **Diet diversity** | **Crop diversity** | **Diet diversity** | **Crop diversity** | **Diet diversity** |
| Household’s wealth | 0.22** (0.07) | 0.24*** (0.06) | 0.18* (0.07) | 0.21*** (0.06) | 0.18* (0.07) | 0.22*** (0.06) | 0.18* (0.07) | 0.22*** (0.06) | 0.17* (0.07) | 0.20*** (0.06) | 0.18* (0.07) | 0.20*** (0.06) |
| Women’s economic Empowerment | 0.03 (0.05) | -0.03 (0.04) | 0.03 (0.05) | -0.02 (0.04) | 0.03 (0.05) | -0.02 (0.04) | 0.03 (0.05) | -0.02 (0.04) | 0.03 (0.05) | -0.02 (0.04) | 0.04 (0.05) | -0.02 (0.04) |
| Total farm size |  |  | 0.15** (0.06) | 0.07 (0.04) | 0.15** (0.06) | 0.07 (0.05) | 0.15** (0.04) | 0.07 (0.05) | 0.16** (0.04) | 0.08 (0.05) | 0.16** (0.06) | 0.08 (0.05) |
| Household size |  |  |  |  | -0.00 (0.04) | -0.04 (0.04) | -0.00 (0.04) | -0.04 (0.04) | -0.01 (0.04) | -0.05 (0.04) | -0.01 (0.04) | -0.05 (0.04) |
| Having off-farm income |  |  |  |  |  |  | -0.00 (0.08) | -0.02 (0.06) | 0.00 (0.08) | -0.01 (0.06) | 0.00 (0.08) | -0.02 (0.06) |
| Respondents finish primary school |  |  |  |  |  |  |  |  | 0.08 (0.05) | 0.13** (0.04) | 0.03 (0.06) | 0.13* (0.05) |
| Age of respondents |  |  |  |  |  |  |  |  |  |  | 0.11* (0.05) | 0.00 (0.04) |
| **R-square** | **0.05** | **0.35** | **0.08** | **0.36** | **0.08** | **0.37** | **0.08** | **0.37** | **0.09** | **0.40** | **0.10** | **0.39** |
| **AIC** | 4265.826 | | 4205.300 | | 4201.382 | | 4205.288 | | 4194.828 | | 4194.341 | |
| **BIC** | 4340.954 | | 4288.524 | | 4292.882 | | 4305.106 | | 4302.965 | | 4310.796 | |

- The SEMs included the `Between village level’ part of the causal model. The `Household-level’ part only includes *`Diet diversity’*, ‘*Crop diversity’* and the variables in the Table. Each explanatory variables is included incrementally. Empty cells indicate unavailable values.
- Models were estimated using Survey round 2
- * p<0.05, ** p<0.01, *** p<0.001

**Table 9:** Standardized effect sizes of `Women’s economic empowerment’ on `Diet diversity’ and ‘Crop diversity’ with unweighted `Market access’ data

| **Effect types** | **First round** | | **Second round** | |
| --- | --- | --- | --- | --- |
|  | **Mean (SE)** | **p-value** | **Mean (SE)** | **p-value** |
| ***Diet diversity*** |  |  |  |  |
| Total effect | -0.125 (0.042) | 0.003 | -0.060 (0.047) | 0.202 |
| Indirect effect | -0.036 (0.017) | 0.032 | -0.029 (0.015) | 0.340 |
| Direct effect | -0.088 (0.039) | 0.024 | -0.031 (0.039) | 0.429 |
| ***Crop diversity*** |  |  |  |  |
| Total effect | 0.018 (0.039) | 0.642 | -0.007 (0.051) | 0.888 |
| Indirect effect | -0.038 (0.014) | 0.007 | -0.040 (0.015) | 0.007 |
| Direct effect | 0.056 (0.037) | 0.126 | 0.032 (0.050) | 0.516 |
